# Viroid Diseases in Pome and Stone Fruit Trees and Koch’s Postulates: A Critical Assessment

**DOI:** 10.3390/v10110612

**Published:** 2018-11-07

**Authors:** Francesco Di Serio, Silvia Ambrós, Teruo Sano, Ricardo Flores, Beatriz Navarro

**Affiliations:** 1 Istituto per la Protezione Sostenibile delle Piante, Consiglio Nazionale delle Ricerche, 70126 Bari, Italy; beatriz.navarro@ipsp.cnr.it; 2 Instituto de Biología Molecular y Celular de Plantas, Consejo Superior de Investigaciones Científicas-Universidad Politécnica de Valencia, 46022 Valencia, Spain; silviaambros@yahoo.es (S.A.); rflores@ibmcp.upv.es (R.F.); 3 Department of Applied Biology and Food Sciences, Faculty of Agriculture and Life Science, Hirosaki University, Hirosaki 036-8561, Japan; sano@hirosaki-u.ac.jp

**Keywords:** viroid pathogenesis, symptoms, woody plants, ADFVd, AFCVd, AHVd, ASSVd, HSVd, PBCVd, PLMVd

## Abstract

Composed of a naked circular non-protein-coding genomic RNA, counting only a few hundred nucleotides, viroids—the smallest infectious agents known so far—are able to replicate and move systemically in herbaceous and woody host plants, which concomitantly may develop specific diseases or remain symptomless. Several viroids have been reported to naturally infect pome and stone fruit trees, showing symptoms on leaves, fruits and/or bark. However, Koch’s postulates required for establishing on firm grounds the viroid etiology of these diseases, have not been met in all instances. Here, pome and stone fruit tree diseases, conclusively proven to be caused by viroids, are reviewed, and the need to pay closer attention to fulfilling Koch’s postulates is emphasized.

## 1. Introduction

Discovery of viroids and inception of the concept of viroid as an infectious minimal RNA, date back to the early seventies of the last century, when potato spindle tuber viroid (PSTVd) [1] and citrus exocortis viroid (CEVd) [2] were identified as the causal agents of the two diseases now associated with their names. Discovery of the first viroids almost seventy years later than that of the first virus (tobacco mosaic virus [3,4]) was likely due to the extremely small size of the former and to their specific structural features being largely different from those of the latter. Viroids are circular RNAs composed of only a few hundred nucleotides (246–434 nt) that, in contrast to viral RNAs, do not code for any protein [5]. As a corollary, viroids accumulate prevalently in vivo as naked RNAs unprotected by tightly-bound host proteins [6,7], and mainly rely on host enzymes and factors for completing their infectious cycle [8]. More than 30 viroid species have been created and classified by the International Committee on Virus Taxonomy (ICTV) in eight genera, which in turn are grouped in two families: *Pospiviroidae* and *Avsunviroidae* [9,10]. Members of the family *Pospiviroidae*: (i) assume a rod-like or quasi rod-like conformation, (ii) localize and replicate in the nucleus, and (iii) contain a characteristic central conserved region (CCR) involved in replication. Members of the family *Avsunviroidae:* (i) fold into rod-like or branched conformations, in some instances stabilized by a kissing-loop interaction; (ii) do not contain a CCR but can form hammerhead ribozymes that mediate self-cleavage of their replicative intermediates of either polarity; and (iii) localize and replicate in plastids, mostly chloroplasts. Viroids infect herbaceous and/or woody plants, in which they may cause severe diseases with relevant economic losses or, alternatively, may go essentially unnoticed (latent) inciting only mild or no symptoms at all [11,12,13,14]. In some cases, disease only results when a viroid co-infects a susceptible host together with a virus, like in grapevine vein banding caused by the concurrent infection of grapevine yellow speckle viroid 1 (GYSVd-1), and/or GYSVd-2, and grapevine fanleaf virus [15]. Thus, the pathogenesis of a viroid is not always obvious and needs specific experimental evidence before being conclusively established.

## 2. Viroid Pathogenicity Requires Fulfilment of Koch’s Postulates

Besides showing the autonomous replication in the absence of any associated helper virus [1,16], T.O. Diener identified the infectious small RNA associated with potato spindle tuber disease as a physical entity by polyacrylamide gel electrophoresis [17]. Moreover, by observing the onset of typical symptoms in an experimental host (tomato) following its inoculation with purified viroid preparations, he also showed the pathogenicity of the infectious RNA [17]. Similar properties were also reported for CEVd, the agent of citrus exocortis disease [2,18,19], thus fulfilling Koch’s postulates as redefined for viruses [20]. Therefore, since the early studies on viroid pathogenesis, and in agreement with the basic concepts developed previously in the study of infectious diseases, the presence of a viroid associated with a specific symptomatology was not considered sufficient for tracing a causal relationship between the former and the latter. Firm evidence demands, whenever possible, to comply with Koch’s postulates (Table 1).

As with viruses, grafting or mechanical inoculation of total RNA preparations from disease-expressing tissues are not appropriate for claiming the etiological role of a viroid present in such preparations, because transmission of other co-infecting agents cannot be ruled out. Therefore, for viroids, as well as for viruses, a key step to fulfil Koch’s postulates is purification of the infectious agent (Table 1). When a viroid RNA accumulates at high levels in the infected plant, its purification is quite easy by a preparative polyacrylamide gel electrophoresis (PAGE) approach [21] specifically aimed at separating the viroid circular form from all other linear RNAs of host or viral origin [22,23]. After electrophoretic separation, ethidium bromide staining and UV irradiation allow visualization of the circular viroid RNA, which can then be gel-eluted and mechanically inoculated into an appropriate host to confirm its autonomous replication and, when applicable, pathogenicity [24,25]. The circular form of some viroids endowed with particularly efficient hammerhead ribozymes, such as chrysanthemum chlorotic mottle viroid (CChMVd), is almost undetectable by these standard electrophoretic methods, most likely due to its self-cleavage during extraction [26]. In this case, more laborious electrophoretic approaches were needed to identify the viroid RNA and show its autonomous replication and pathogenicity [26,27]. Woody hosts, like pome and stone fruit trees, may pose additional problems in viroid purification because the abundant polysaccharides present in crude nucleic acid preparations, obtained by standard extraction and fractionation procedures, then interfere with PAGE separation of RNAs. Although removal of most polysaccharides can be achieved by further extracting RNA solutions with 2-methoxyethanol in phosphate buffer [28], viroid purification can still remain difficult when the accumulation level is very low in the natural host and there is no alternative experimental host, thus becoming the limiting factor for complying with Koch’s postulates.

To overcome this problem, it is recommended to clone dimeric head-to-tail cDNAs of the full-length viroid RNA into plasmid vectors, which can be amplified in *Escherichia coli*, purified and used directly or serve for in vitro transcription of viroid RNAs (Table 1); full-length monomeric cDNAs or their transcripts are also infectious, but significantly less than their dimeric counterparts [29]. This approach generates high amounts of the desired transcripts to be then bioassayed for infectivity and pathogenicity. An alternative is to transform a disarmed *Agrobacterium tumefaciens* strain with a Ti plasmid containing dimeric head-to-tail viroid cDNAs. Infiltration of the transformed agrobacterium (agroinoculation) into the host may allow the transient expression of the viroid RNA within the cells and triggering of replication and systemic infection (Table 1). In this respect, it is worth noting that a viroid RNA purified from an infected host and an in vitro- or agrobacterium-generated viroid RNA are not in principle equivalent, especially when it comes to pathogenicity. Indeed, while viroids accumulate in infected plants as quasispecies composed of populations of sequence variants differing in a few nucleotide positions [29,30,31,32] viroid RNAs transcribed in vitro or agroexpressed in vivo are composed of a single sequence variant whose pathogenicity may differ from that of the natural viroid population from which the viroid cDNA was cloned (see below). Particularly pertinent to the main theme of this article is to highlight that the conclusions derived from inoculations with in vitro-generated transcripts and/or agroinoculation are compelling, because the infectious agent is actually generated *ex novo*, excluding the possibility that another agent present in the original host (i.e., a virus or co-infecting viroid) could be accidentally co-transmitted.

Although several viroids have been reported from symptomatic pome and stone fruits, their direct involvement in causing a specific disease (complying with Koch postulates) has only been achieved in some instances. For viroids inducing symptoms on fruits or bark, the time elapsing between inoculation and symptom expression is generally long (from months to years). This time interval, however, can be shortened significantly by back-inoculating chips from seedlings infected with the purified or clonally amplified viroid RNA onto adult trees (i.e., already producing fruits) previously tested as free of known viroids, viruses and phytoplasma. Even if the possibility that an unknown infectious agent could be present in the viroid-infected seedlings or in the grafted adult trees, cannot be completely dismissed, such a risk is considered minor and symptoms expressed in fruits (or in bark) of the adult tree are assumed as caused by the viroid infecting the original inoculated seedlings (Table 1).

In the following sections, disorders affecting pome and stone fruits attributed to viroids have been re-evaluated following careful examination of the original literature (Table 2), with the focus then put on those for which the involvement of a viroid as the causal agent of the disease has been formally verified by fulfilling Koch’s postulate.

## 3. Viroid Diseases Caused by Apple Scar Skin Viroid (ASSVd)

*Apple scar skin viroid* is the type species of the genus *Apscaviroid* (family *Pospiviroidae*) [9]. Characterization of sequence variants from naturally infected trees showed that ASSVd consists of 329–334 nt [33,49,50]. This viroid is the etiological agent of two apple diseases: apple scar skin and dapple apple.

**Figure 1:**
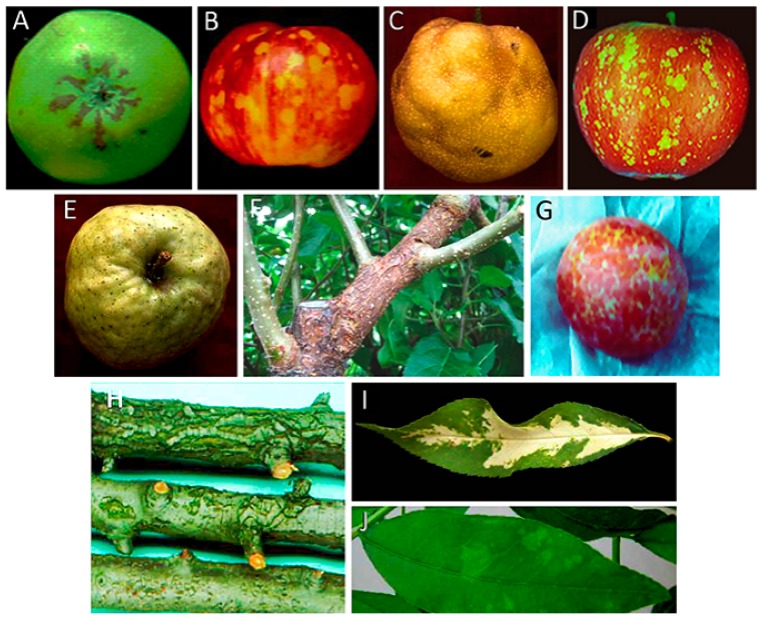
(**A**) Symptoms induced by ASSVd on apple fruits cv. ‘Indo’ (Courtesy of J.C. Desvignes); (**B**) symptoms induced by ASSVd on apple fruits cv. ‘Starkrimson’ (Courtesy of J.C. Desvignes); (**C**) symptoms of pear fruit dimple induced by ASSVd on Japanese pear; (**D**) symptoms induced by ADFVd on apple fruits cv. ‘Starkrimson’ (Courtesy of J.C. Desvignes); (**E**) symptoms of fruit crinkle on apple fruit cv. ‘Orin’ induced by AFCVd; (**F**) symptoms of blister bark on cv. ‘Nero 26′ induced by AFCVd (Courtesy of D. Ito); (**G**) symptoms of dapple induced by HSV in plum (Courtesy of H.L. Sänger); (**H**) symptoms induced by PBCVd in pear cv. ‘A 20′ (Courtesy of J.C. Desvignes); (**I**) symptoms of severe albinism (calico) induced by PC-40 variant of PLMVd in leaves of the peach seedling GF-305; (**J**) symptoms of green mosaic induced by gds3 variant of PLMVd in leaves of the peach seedling GF-305.

Apple scar skin disease was first reported in China and Japan in the 30s and 50s of the last century, respectively, where it caused important economic losses [51]. The disease symptoms in apple fruits consist of brownish scar-like lesions (Figure 1A). A small circular RNA with the typical features of a viroid was associated with symptomatic plants. Inoculation of young apple seedlings with this PAGE-purified viroid-like RNA and its subsequent detection in the inoculated plants after a few months showed autonomous replication and systemic trafficking. However, no pathogenicity could be associated with this viroid due to the absence of fruits in the inoculated seedlings [52,53]. Further observation of apple scar skin symptoms in the inoculated plants and sequencing of this RNA, thereafter named ASSVd, confirmed the viroid etiology of the disease [33].

Dapple apple disease, first reported in USA around the 50s of the previous century [54] and later on in Canada, Japan, China, UK and South Korea [50], is characterized by fruit symptoms consisting of small circular greenish spots that may coalesce into large discolored areas (Figure 1B). The typical spots are generally more abundant on the calix area of the fruits. This disease was initially attributed to the same causal agent of apple scar skin disease [55] and, after molecular characterization, to the same viroid [38]. More specifically, ASSVd variants of 331 nt were found to be closely associated with dapple apple symptoms [34]. Successful systemic infection of apple and pear seedlings was obtained by agroinoculation of a dimeric head-to-tail cDNA of ASSVd isolated from fruits affected by dapple apple disease [35]. Molecular analysis of the viroid progeny from the infected seedlings confirmed that the ASSVd recovered was identical to the inoculated variant, thus conclusively confirming its infectivity [35]. In addition, inoculation with chips from an apple tree only known to be infected with the PK13 isolate of ASSVd triggered dapple apple and/or scar skin symptoms in several apple cultivars 2 years after inoculation [56], thus further confirming the involvement of the same viroid in both apple diseases.

Although ASSVd is latent in most pear species inoculated experimentally [56], a fruit disorder observed in the Japanese pear cvs. ‘Niitaka’ and ‘Yoshino’ consisting of dimples in the mature fruit surface (Figure 1C) [57], is caused by ASSVd. To fulfill Koch’s postulates, pear seedlings were first inoculated with the gel-purified viroid RNA from a pear tree showing fruit dimpling. In parallel experiments, pear seedlings were inoculated with purified ASSVd isolated from apple trees expressing scar skin symptoms. Material from these seedlings was used to graft-inoculate viroid-free adult Japanese pear trees of cvs. ‘Niitaka’ and ‘Yoshino’, which 3 years later displayed dimpling symptoms in their fruits independently of whether they were inoculated with the viroid RNA purified from pear or apple. Moreover, a viroid RNA with the same electrophoretic mobility as ASSVd was detected by return-PAGE in the trees showing dimple fruit symptoms and its molecular characterization revealed that in all cases it was an ASSVd variant [36]. These results confirmed that ASSVd is also the etiological agent of this disease, named Japanese pear fruit dimple.

Other pear fruit disorders, like pear rusty skin [34,38] and pear fruit crinkle diseases reported in China [39], and scarred, cracked or russeted pear fruits reported in Greece [40,41] have been associated with ASSVd and/or with peach latent mosaic viroid (PLMVd) (see below). However, confirmation of the viroid etiology of these diseases still awaits fulfillment of Koch’s postulates.

ASSVd has been also reported to naturally infect apricot, peach, sweet cherry [58,59,60] and Himalayan wild cherry [61], but no information on related diseases and/or on the epidemiological relevance of these findings is available. A comprehensive review on the molecular and biological features of ASSVd and its distribution, transmission, detection and control has been recently published [50].

## 4. Viroid Disease Caused by Apple Dimple Fruit Viroid (ADFVd)

Apple dimple fruit disease, a disorder characterized by malformed apple fruits showing roundish and depressed green areas on the red skin (Figure 1D), was observed in Southern Italy in the apple cv. ‘Starking delicious’ [62]. The symptoms were similar to those of dapple apple caused by ASSVd in some apple varieties. The identification and molecular characterization of a small RNA with the structural characteristics typical of a viroid, but significantly different in sequence from ASSVd, together with its close association with symptomatic apple fruits, suggested the putative viroid aetiology for the disease [62]. Such hypothesis was confirmed a few years later when the ability of ADFVd to infect and induce symptoms was experimentally shown: After inoculation of purified ADFVd forms in young apple seedlings, this viroid was detected in most of the inoculated plants, thus proving its infectivity. Moreover, the observation of the typical symptoms on the fruits of several apple cultivars, graft-inoculated using material from the ADFVd-infected seedlings, confirmed that this viroid was the causal agent of apple dimple fruit disease [24]).

ADFVd isolates, composed of sequence variants ranging in size from 306–307 nt, have been characterized in Italy, China, Lebanon and Iran [62,63,64,65,66], while an isolate containing divergent variants of 303 nt has been reported in Japan [67]. Phylogenetic analyses of the ADFVd variants have shown that they cluster according to their geographic origin [68]. Recently, a variant of ADFVd has been reported from fig in Italy [68], but no data about the pathogenicity of the viroid in this host are available. ADFVd belongs to the genus *Apscaviroid* within the family *Pospiviroidae* [9]. Other biological and molecular features of ADFVd have been recently reviewed [69].

## 5. Viroid Diseases Caused by Apple Fruit Crinkle Viroid (AFCVd)

Variants of this viroid have 369 to 372 nt and 85–75% sequence identity with Australian grapevine viroid (AGVd) [70], which belongs to the genus *Apscaviroid* 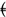
family *Pospiviroidae*) [9]. AFCVd has not been yet classified as a novel species since no discriminating biological traits have been reported with respect to AGVd.

Apple fruit crinkle, a graft-transmissible disease found so far only in Japan [71], is characterized by crinkling and dappling of the mature fruit surface (Figure 1E). Initially, the disease was found associated with a viroid-like RNA with higher size and different sequence than ASSVd. The infectivity of such an RNA, and therefore its viroid nature, was verified by transmission of the gel-purified forms to apple seedlings [72]. However, these bioassays did not disclose whether the viroid was the agent of the disease, because the inoculated seedlings did not develop any leaf or bark symptoms, and they did not produce fruits during the analysis [72]. Graf-inoculations, using chip-buds from the apple seedlings previously infected with the purified AFCVd, showed that this viroid incites crinkling and necrosis in the fruit flesh of cv. ‘Ohrin’, and crinkle and dapple symptoms in fruits of cv. ‘Jonathan’ [37]. In the same study, after AFCVd inoculation, apple trees of cvs. ‘Starking Delicious’ and ‘Nero 26’ developed severe blister bark symptoms on trunks and branches very similar to those reported previously in cv. ‘Nero 26’ in Japan [73] (Figure 1F), strongly suggesting the involvement of AFCVd also in this bark disorder [37]. Based on these findings, a viroid-like RNA of size similar to AFCVd was isolated by bi-directional PAGE analysis from cv. ‘Nero 26’ showing severe blister bark symptoms. The nucleotide sequence of this viroid-like RNA completely matched that of the reference isolate of AFCVd (P-196). The gel-purified AFCVd RNA was successfully transmitted by razor slashing to apple seedlings that were used as source material for grafting two ‘Nero 26’ trees. Three years later, the latter turned positive to AFCVd infection and showed blister bark symptoms on the branches similar to those observed on the original ‘Nero 26’ tree [42]. These experiments fulfilled Koch’s postulates, thus confirming that this viroid is the causal agent of blister bark disease of apple cv. ‘Nero 26’.

More recently, AFCVd has been detected in hop showing stunting and leaf curling [74] and in Japanese persimmon [75]. While Koch’s postulates confirming that this viroid is the causal agent of a hop disorder similar to that incited by hop stunt viroid have been fulfilled [74,76], the association of AFCVd with disease in persimmon remains unclear. Additional information on the biology, spread and control of AFCVd has been presented in a recent review [69].

## 6. Viroid Disease Caused by Pear Blister Canker Viroid (PBCVd)

This viroid, with a genome of 315 nt, has been classified into the genus *Apscaviroid* (family *Pospiviroidae*) [77]. Several bark disorders on certain pear cultivars had been described in Europe and USA, for which a common infectious agent was initially proposed [78]. However, with the identification of PBCVd associated with only one of these disorders [79], the situation appeared more complex, suggesting the likely involvement of different causal agents. Pear blister canker (PBC) was the name assigned to a disease characterized by bark alterations in the pear cv. ‘A 20’, consisting of pustules or superficial cracks that during the second year of infection turned into cankers and scaly bark (Figure 1H), with no alterations being expressed in leaves and fruits [80]. Later on, a viroid etiology for this disease was proposed, since a small RNA with the characteristic structural properties of viroids was identified in pear trees infected with an agent, inducing PBC symptoms in the pear cv. ‘A 20’. Moreover, accumulation of the small circular RNA, then tentatively called PBCVd, in cucumber and pear seedlings inoculated with the electrophoretically-purified viroid-like RNA, showed its ability to replicate and traffic autonomously [81]. Since PBC symptoms are not expressed in young pear seedlings, proof of PBCVd pathogenicity was not achieved at that time. Further bioassays propagating the pear cv. ‘A 20’ onto the pear seedlings previously inoculated with PBCVd, resulted in the expression of PBC symptoms 2 years later in the indicator, from which PBCVd was recovered, thus fulfilling Koch’s postulates [43]. Since the symptoms induced by PBCVd take so long to develop in the pear cv. ‘A 20’, an alternative indicator was searched. Examination of a number of seedlings of the perry pear ‘Fieudière’ resulted in the identification of two selections particularly susceptible to PBCVd displaying petiole, leaf and bark necrosis 3 to 5 months after inoculation [82].

It is worth noticing, that while PBCVd causes symptoms in the two above-mentioned indicators, it is latent in most commercial pear cultivars [82]. Actually, almost 10% of the old French varieties are infected latently by PBCVd [43]. Since PBC symptoms can be confused with those incited by fungi or bacteria, a specific test to detect PBCVd is required for evaluating the incidence of the disease. PBCVd and issues related to its biology, spread, control and detection have been recently reviewed [69].

## 7. Viroid Diseases Associated with Hop Stunt Viroid (HSVd)

The genome of HSVd, genus *Hostuviroid* (family *Pospiviroidae*), consists of 294–303 nt. This viroid, first identified in hop showing stunting and leaf curling [83], can naturally infect a wide number of host species, including stone and pome fruit trees, being latent in most of them [84]. However, in some others, such as cucumber, citrus, peach and plum, HSVd causes or has been associated with important diseases [84]. After the first complete genome sequence of HSVd was determined [85], a high number of sequence variants were reported clustering in essentially five phylogenetic groups according with the natural hosts in which they were identified (hop, citrus, plum-grape, plum-citrus and plum-hop-cit3) [86,87].

Plum dapple fruit is a grafted-transmissible disease, first reported in Japan [88], which was associated with a viroid-like RNA having a size and sequence similar to HSVd [45,89]. The disease, characterized by red blotches in plum fruits (Figure 1G), resembles the yellowish red alterations observed in fruits of plum cv. ‘Soldam’ [90], with the two diseases proposed to have the same etiological agent according to graft-transmission assays. Moreover, typical symptoms of dapple fruits were observed on branches of plums grafted with buds collected from a plum seedling inoculated with a highly purified HSVd preparation from a symptomatic plum tree [44], thus showing the direct involvement of this viroid in the plum disease. On the other hand, chlorotic blotches on peach fruits, denoted as peach dapple fruit, were also first reported in Japan, and are associated with HSVd infection [45]. Inoculation of cucumber plants with purified preparations of HSVd from plum and peach dapple fruits induced stunting, leaf curling and vein clearing, the typical symptoms of HSVd in this herbaceous host [45]. Comprehensive reviews on HSVd have been published [84,91].

## 8. Viroid Diseases Caused by Peach Latent Mosaic Viroid (PLMVd)

This viroid belongs to the family *Avsunviroidae* and is the type member of the genus *Pelamoviroid* [10]. The PLMVd reference variant has a size of 338 nt [92], but many other sequence variants have been characterized with sizes ranging from 335 to 351 nt. PLMVd RNAs of either polarity are endowed with self-cleaving activity mediated by hammerhead ribozymes, and adopt multi-branched secondary structures [92,93,94,95], which in the (+) strand is stabilized by a kissing-loop interaction [96,97]. Molecular and biological aspects of PLMVd have been reviewed previously [98,99,100]. Here, we will focus on the evidence supporting the direct involvement of PLMVd in certain disorders, so far affecting only peach.

Diseases termed peach calico (PC) [101], peach blotch (PB) [102], peach yellow mosaic (PYM) [103] and peach latent mosaic (PLM) [104], were initially reported in the United States (the first two), Japan and France, respectively. They are characterized by leaf chloroses of different severity, ranging from albinism (PC) (Figure 1I) to greenish patches (PB) and yellow or mild green mosaics (Figure 1J) (PYM and PLM, respectively), often associated with discolorations in stems and fruits (which additionally display deformations and suture cracking), delays in flowering and ripening, and alteration of the tree growing pattern (open habit). The term latent in PLM does not indicate absence of symptoms, but rather refers to the long time required for their expression in the field [98].

Based on cross-protection assays between different isolates [104,105], these four disease were suspected to be caused by a related infectious agent. The identification of a small circular RNA associated with PLM led to the proposal of a viroid etiology for this disease [106], a view further confirmed when: (i) GF-305 peach seedlings inoculated with preparations of this viroid-like RNA purified from a severe PLM isolate expressed the characteristics symptoms, and (ii) an RNA with the same physical properties (now a *bona fide* viroid) was retrieved from the symptomatic tissue [46]. These experiments fulfilled Koch’s postulates, thus verifying that PLMVd is the etiological agent of PLM. Moreover, sequencing of PLMVd [92], allowed development of molecular probes that identified this viroid in peach trees showing PC and PYM, thus supporting they were different manifestations of the same disorder [47,79].

Studies covering a period of almost 20 years have addressed the question of how the same pathogen can elicit different diseases. Molecular characterization of three phenotypically different PLMVd field isolates and inoculation of in vitro-generated infectious transcripts of specific cDNA clones, have disclosed a correlation between the pathogenicity of the PLMVd isolates and both the complexity of the viroid populations and the presence of specific sequence variants [29]. Further studies have identified variants that induce reproducible leaf mosaics in the inoculated seedlings (i.e., variant gds6), more variable phenotypes (i.e., variant gds15) or no symptoms at all (i.e., variants esc10 and ls11) [93]. Altogether, these data pointed to the existence of variants with pathogenic determinant(s) responsible for each specific symptom, although the complexity of their progeny accumulating in the inoculated seedlings hampered conclusive identification of such determinants [29,93].

A significant advance in solving this conundrum resulted from the molecular and biological characterization of PLMVd variants associated with PC [47]. This study first revealed a close association of the albino phenotype with variants containing an insertion of 12–14 nt, folding into a hairpin capped by a U-rich loop, located at the end of the hammerhead arm in the proposed PLMVd branched secondary structure. Then, inoculation of in vitro-generated dimeric transcripts of PLMVd variants containing this structural element, appearance of the albino phenotype in the inoculated seedlings, and recovery of variants with the same pathogenic determinant, fulfilled Koch’s postulates [47]. These results were further confirmed by parallel experiments in which no symptoms were observed in seedlings inoculated with a variant from which the pathogenic determinant was removed by site-directed mutagenesis [47], and by another study with two different PC isolates, in which additional mutagenesis and bioassays further supported the pathogenicity of variants containing the identified PC determinant [107]. Moreover, these findings also showed that the PC determinant can be acquired and lost [47] and, importantly, that PLMVd variants containing such a determinant accumulate preferentially in albino sectors of PC-expressing leaves [107].

In PC, a combination of ultra-structural, biochemical, and molecular analyses identified an early step of the chloroplast developmental program as that specifically compromised by PLMVd variants with the PC determinant [108]. Moreover, deep sequencing of the viroid-derived small (21–24 nt) RNAs (vd-sRNAs) accumulating in infected tissues, semi-quantitative RT-PCR and RNA ligase-mediated rapid amplification of cDNA ends (RACE), showed that two 21-nt vd-sRNAs containing the pathogenic determinant of the PC-inducing variants—likely generated by a cellular Dicer-like enzyme—target for cleavage, via RNA silencing, the host mRNA coding for the chloroplastic heat-shock protein 90 (cHSP90), the homologue of which, in arabidopsis, mediates chloroplast biogenesis [109]. More recent studies have shown that a similar mechanism operates in an intense PYM incited specifically by variants containing a completely different pathogenic determinant, with the corresponding vd-sRNA holding this determinant targeting for cleavage the host mRNA coding for another chloroplastic protein [48]. Other PLMVd variants, likely bearing different pathogenic determinants, have been associated with yellowing and chlorosis along leaf edges, although the targeted host mRNAs have not been identified [110].

## 9. Concluding Remarks

Due to their small size and circularity, viroid genomic RNAs can be easily purified from infected plants and used for testing their autonomous replication and pathogenicity. This approach is particularly feasible when viroids accumulate at relatively high titer in the infected host. When purification is hampered by the low titer of the viroid or by difficulties in extracting it from hard ligneous tissues, infectious viroid RNAs can be generated in vitro or in vivo. Therefore, fulfillment of Koch’s postulates appears now feasible for most viroid-host combinations, even if this task is time-consuming for tree diseases characterized by fruit or bark symptoms that may take years to develop. This is the main reason explaining why the postulates have not (yet) been met in several fruit tree diseases that have been associated for a long time with viroid infections. A recent case is apple hammerhead viroid (AHVd), which was first reported as a viroid-like RNA in 2014 in China [111]. Confirmation of the genuine viroid nature of this entity was provided recently by bioassays with in vitro-generated viroid transcripts [112]. No symptoms were observed in the inoculated plants, thus suggesting that AHVd is latent, at least in the apple cultivars used in the bioassay. However, since this viroid has been found also in naturally-infected Canadian apple trees of cv. ‘Pacific Gala’ showing swelling and radial limb cracking [113], additional bioassays run for longer times are needed to exclude that AHVd may induce symptoms only in some specific cultivars [111]. This example illustrates how obtaining conclusive evidence on the pathogenicity of viroids infecting woody plants, such as pome or stone fruit trees, may be sometimes demanding. These difficulties, however, should not discourage researchers to pursue solid evidence, because a compelling answer on the pathogenicity of a viroid, or any other infectious agent, is of primary relevance to assess whether it is a pest and to address other issues on phytosanitary risks. Such considerations appear particularly appropriate nowadays, when the expanding use of high-throughput sequencing technologies have already allowed the identification of known and novel viroids or viroid-like RNAs in several plants [110,113,114,115,116], with additional viroids expected to be found in the near future in symptomatic and symptomless hosts.

## Figures and Tables

**Table 1:** Koch’ postulates for providing evidence of the involvement of a viroid in a plant disease *.

1. The viroid must be concomitant with the disease
2. The viroid must be: -isolated from the diseased plant and/or generated *ex novo*, either in vitro (RNA transcription) or in vivo (agroinoculation)
-multiplied in the original and/or in an experimental host
-purified physico-chemically (i.e., by electrophoresis)
-identified for its intrinsic properties (i.e., circularity, size and sequence)
3. When the purified viroid or the corresponding RNAs generated *ex novo* are inoculated into a healthy host plant, they must reproduce the disease
4. The same viroid must be re-isolated from the inoculated natural and/or experimental host

* Modified and adapted from Table 4 of Bos (1981) [20].

**Table 2:** Pome and stone fruit diseases caused by or associated with viroids.

Disease	Host	Viroid	Koch’s Postulates	Reference
Apple scar skin	Apple	Apple scar skin viroid	Yes	[33]
Dapple apple	Apple	Apple scar skin viroid	Yes	[34,35]
Japanese pear fruit dimple	Pear	Apple scar skin viroid	Yes	[36]
Apple fruit crinkle	Apple	Apple fruit crinkle viroids	Yes	[37]
Pear rusty skin	Pear	Apple scar skin viroid	No	[34,38]
Pear fruit crinkle	Pear	Apple scar skin viroid	No	[39]
Scarred, cracked, russeted pear fruit	Pear	Apple scar skin viroid/peach latent mosaic viroid	No	[40,41]
Apple dimple fruit	Apple	Apple dimple fruit viroid	Yes	[24]
Blister bark in cv. ‘Nero26’	Apple	Apple fruit crinkle viroid	Yes	[42]
Pear blister canker in cv. ‘A20’	Pear	Pear blister canker viroid	Yes	[43]
Plum dapple fruit	Plum	Hop stunt viroid	Yes	[44]
Peach dapple fruit	Peach	Hop stunt viroid	No	[45]
Peach latent mosaic	Peach	Peach latent mosaic viroid	Yes	[46]
Peach calico	Peach	Peach latent mosaic viroid	Yes	[47]
Peach yellow mosaic	Peach	Peach latent mosaic viroid	Yes	[48]
